# Complete genome sequence of *Peribacillus* sp. S4 isolated from the biofilter

**DOI:** 10.1128/mra.01356-24

**Published:** 2025-04-30

**Authors:** Dan Wu, Dongfang Meng, Yang Wang, Yongjian Piao, Yixing Yuan, Fugui Zhang, Lixia Zhang, Shuang Zhang

**Affiliations:** 1 Longjiang Environmental Protection Group Co., Ltd 47822, Harbin, China; 2 School of Environment, Harbin Institute of Technology 547112, Harbin, Heilongjiang, China; University of Southern California, Los Angeles, California, USA

**Keywords:** *Peribacillus*, complete genome sequence, nitrogen metabolism

## Abstract

We report the complete genome of *Peribacillus* sp*.* S4. The results showed that the genome of strain S4 consisted of a 5,582,479 bp chromosome with a GC content of 40.49% and encoded 5,087 genes. Genes such as *NRT*, *narK*, *nrtP*, and *nasA* related to nitrogen metabolism were annotated. In addition, genes involved in nitrogen assimilation pathways were identified, including *gltB*, *glnA*, and *gudB*. This work will be valuable for further study on the possible role of strain S4 in the bioconversion of nitrogen in composting.

## ANNOUNCEMENT

The genus *Peribacillus*, formerly part of the *Bacillus* genus, is a newly discovered taxon (1). Some species of the genus *Bacillus* can reduce ammonia emissions during composting (2). However, the application of the genus *Peribacillus* in composting was poorly reported. The sawdust sample was taken from the surface filler of the biofilter of Harbin sludge composting plant, Heilongjiang Province, China (45°48′48″ N 126°42′51″ E) and was shaken for 48 h (30°C, 160 rpm) in an enrichment medium, containing sucrose (50.0 g/L), KH_2_PO_4_ (2.0 g/L), NaCl (2.0 g/L), MgSO_4_ (0.5 g/L), FeSO_4_ (0.1 g/L), and ZnSO_4_ (0.5 g/L), supplemented with ammonia (10 mL/L). Subsequently, the enriched solution underwent serial dilution. The diluted sample was spread onto an LB agar plate containing 0.5% ammonia. After incubation at 30°C for 3 days, colonies were carefully selected and further purified through streaking on LB agar plate. The strain was subcultured four times on an LB agar plate. Finally, a single strain, designated as S4, was isolated.

DNA was isolated from a monoculture of strain S4 cultivated in beef extract peptone liquid medium (100 mL) for 2 days (30°C, 160 rpm) using E.Z.N.A Bacterial DNA Kit (Omega, Norcross, GA, USA). The same DNA extraction was used for both PacBio and Illumina libraries. The complete genome was sequenced using the Illumina NovaSeq 6000 and the PacBio Sequel II by Shanghai Biozeron Biotechnology Co., Ltd., Shanghai, China. Highly qualified DNA sample (OD_260/280_ = 1.8–2.0, >6 µg) was utilized to construct an Illumina library by the TruSeq Nano DNA Sample Prep Kit (Illumina), generating 3,470,000 2 × 150 bp length reads. For PacBio, libraries were prepared with the Express Template Prep Kit 2.0 (PacBio, CA, USA). DNA was sheared by a Megaruptor 3 (Diagenode, USA), and size selection was performed using the BluePippin size selection system (cutoff value: <8 kb). The number of reads is 1,233,128, and the raw read *N*_50_ is 2,245 bp for PacBio. For quality control, reads containing ≤90% of bases at Q30 were filtered out to remove low-quality data. Adapter trimming and read filtering were performed using Trimmomatic version 0.36 (3) (ILLUMINACLIP:Adapter:fasta:2:30:10, SLIDINGWINDOW:4:15, MINLEN:75). Genomes were assembled with Unicycler version 0.5.0 (4) and were annotated by PGAP version 6.9 (5). tRNA was identified using the tRNAscan-SE version 2.0.4 (6), and rRNA was determined using the RNAmmer version 1.2 (7). The genome was circularized by circlator version 1.5.5 (8). Average nucleotide identity (ANI) was analyzed by Pyani version 0.2.11 (9). Default parameters were used for all software unless otherwise specified.

Strain S4 consisted of a chromosome of 5,582,479 bp (40.49% GC content and a coverage of 186×) and encoded 5,087 genes. A total of 83 tRNAs and 45 rRNAs were identified. ANI analysis showed 98.39% similarity to *Peribacillus frigoritolerans* CP091882 with the ANI values of 98.39%. Therefore, strain S4 was classified as the genus *Peribacillus*. Genes such as *NRT*, *narK*, *nrtP*, and *nasA* related to nitrogen metabolism were annotated. Genes involved in nitrogen assimilation pathways were identified, including *gltB*, *glnA*, and *gudB*. This work will be valuable for further study on the possible role of strain S4 in the bioconversion of nitrogen in composting.

## Data Availability

The whole-genome sequence has been deposited in the NCBI GenBank database under accession number CP175708, BioProject accession number PRJNA1193798, and BioSample accession number SAMN45155002. The raw sequences were deposited under the accession number SRR31605586.
